# *Notes from the Field: Mycobacterium abscessus* Outbreak Related to Contaminated Water Among Ventilator-Dependent Residents of a Pediatric Facility ― Pennsylvania, 2022

**DOI:** 10.15585/mmwr.mm7242a5

**Published:** 2023-10-20

**Authors:** Jenna N. Sinkevitch, Julie Paoline, Aaron Smee, Sophie Jones, Kevin Spicer, Paige Gable, Hollis Houston, Valerie Stevens, Cara Bicking Kinsey

**Affiliations:** ^1^Bureau of Epidemiology, Pennsylvania Department of Health; ^2^Council of State and Territorial Epidemiologists, Atlanta, Georgia; ^3^Division of Healthcare Quality Promotion, National Center for Emerging and Zoonotic Infectious Diseases, CDC; ^4^Epidemic Intelligence Service, CDC.

*Mycobacterium abscessus*, a nontuberculous mycobacterium found in water and soil, is an opportunistic pathogen responsible for waterborne illness outbreaks in health care settings (*1*). On September 29, 2022, the Pennsylvania Department of Health (PADOH) received notification of *M. abscessus*–positive respiratory isolates from ventilator-dependent residents of a 34-bed pediatric facility. The facility is licensed for residential services, but not as a health care facility. A case was defined as the first *M. abscessus*–positive culture identified from a resident of this facility during March–August 2022. Three cases were identified: two colonizations and one clinical infection. PADOH investigated this outbreak to identify risk factors and recommend infection prevention and control (IPC) measures.

## Investigation and Outcomes

On October 12, PADOH conducted a site visit to observe IPC practices. Three instances of respiratory care were observed, during which respiratory therapists failed to follow aseptic technique. Of 37 observed opportunities for hand hygiene, 30 (81%) were compliant. With regard to tracheostomy tube reprocessing (i.e., cleaning and disinfection for reuse), the manufacturer provided different instructions for home care and health care settings; the latter included stricter processes to reduce the risk for pathogen transmission. Staff members did not follow either of the manufacturer’s processes, and instead, created their own procedure using nonmedical cleaning tools and an ultraviolet baby bottle sterilizer for which there is no documentation of effectiveness. PADOH recommended that the facility follow the more stringent manufacturer instructions for tracheostomy tube reprocessing for health care settings* and use aseptic practices during respiratory care. A second site visit on October 31 showed improvement in IPC practices; however, staff members were still not reprocessing tracheostomy tubes following manufacturer recommendations for health care. This activity was reviewed by CDC, deemed not research, and was conducted consistent with applicable federal law and CDC policy.^†^

Multiple areas of the building had not been in use for 2 years, raising concern about stagnant water and contamination. The facility did not have a water management plan or monitor water quality, as is recommended to prevent water-related infections (*2*). On October 31, PADOH collected 21 environmental samples from faucets, aerators, showerheads and drains, filtered water and ice machines, and water samples from clinical care areas where residents with *M. abscessus* colonization or infection resided. *Mycobacterium* species were identified in 16 (76%) of 21 environmental samples; *M. abscessus* was identified from a shower drain swab (Table). Heterotrophic plate counts of paired first-catch and post-flush^§^ water samples in a room sink of a resident and the reprocessing room sink where tracheostomy tubes were reprocessed exceeded Environmental Protection Agency (EPA) standards for safe drinking water^¶^ (*3*). PADOH recommended that the facility hire a water management consultant, develop a water management program, and install point-of-use filters in clinical areas until water quality consistently met EPA standards. The facility implemented recommendations in collaboration with a water management consultant; PADOH continued collaborative monitoring for an 8-month period. 

**TABLE:** Organism growth and heterotrophic plate count* results for environmental specimens collected from water sources in a pediatric facility — Pennsylvania, 2022

Sampling site	Sample type	NTM identified	Heterotrophic plate counts (CFU/mL)^†^
Resident 1 room sink	First-catch water	*Mycobacterium franklinii*; *M. chelonae*	25,000
	Post-flush water	*M. franklinii*; *M. chelonae*	7,800
	Faucet swab	*M. gordonae*	NP
Tracheostomy tube reprocessing room sink	First-catch water	*M. chelonae*; *M. llatzerense*; *M. franklinii*	9,600
	Post-flush water	*M. llatzerense*	5,500
	Faucet swab	No NTM growth^§^	NP
Resident 2 room sink	Faucet swab	*M. gordonae*	NP
	Aerator swab	No suspected growth	NP
Filtered water machine	First-catch water	*M. chelonae*; *M. llatzerense*	NP
	Spout swab	*M. gordonae*	NP
Stand-alone shower in shower room	First-catch water	*M. llatzerense*	NP
	Faucet swab	No NTM growth	NP
	Drain swab	*M. chelonae*; *M. abscessus*	NP
Multipurpose room sink	First-catch water	*M. franklinii*; *M. llatzerense*	NP
	Faucet swab	*M. gordonae*	NP
Hallway handwashing sink	First-catch water	*M. llatzerense*	NP
	Faucet swab	*M. gordonae*	NP
Ice machine	Ice	*M. peregrinum*; *M. llatzerense*	NP
	Ice guard swab	No NTM growth	NP
	Chute swab	No NTM growth	NP
	Drain swab	No NTM growth	NP

**Abbreviations**: CFU = colony-forming unit; HPC = heterotrophic plate count; NP = not performed; NTM = nontuberculosis mycobacterium.

* HPCs measure carbon-consuming microorganisms in water and can be used to assess conditions affecting microbial growth in a water source or distribution system. HPCs were performed on paired first-catch and post-flush water samples only. HPCs were not performed on swab samples.

^†^ HPCs are reported as CFUs per mL and provide an estimate of the total number of viable organisms in a sample. Environmental Protection Agency standards for safe drinking water are HPC ≤500 CFU/mL.

^§^ Selective medium was used to culture and isolate the target organism or species, and no presumptive NTM growth was found in the environmental sample.

## Preliminary Conclusions and Actions

Epidemiologic and laboratory evidence suggest that this outbreak of *M. abscessus* was related to substandard water quality and inadequate IPC practices. Extended disuse of space in the building potentially resulted in stagnant water in plumbing, and the lack of a water management program meant that water quality was unmonitored. The facility licensing requirements did not emphasize IPC standards necessary for residents with high medical needs, including those who are dependent on ventilators. In ventilator-capable congregate settings IPC-recommended procedures should meet health care standards to prevent transmission of infectious organisms. 

## Footnotes

* https://8949755.fs1.hubspotusercontent-na1.net/hubfs/8949755/User%20Manual/Bivona%20TTS%20Neonatal%20and%20Pediatric%20Trach%20Tube%20Instruction%20PKG-DFU-PNT-2%20Rev004%2009_13%20(007).pdf

† 45 C.F.R. part 46, 21 C.F.R. part 56; 42 U.S.C. Sect. 241(d); 5 U.S.C. Sect. 552a; 44 U.S.C. Sect. 3501 et seq.

§ First-catch samples were collected before aerator removal and occurred before the water outlet being sampled was used. Post-flush samples were collected after letting the water run for 5 minutes.

¶ EPA standards for safe drinking water are a heterotrophic plate count ≤500 colony-forming units/mL.
